# Transient Increases in Neural Oscillations and Motor Deficits in a Mouse Model of Parkinson’s Disease

**DOI:** 10.3390/ijms25179545

**Published:** 2024-09-02

**Authors:** Yue Wu, Lidi Lu, Tao Qing, Suxin Shi, Guangzhan Fang

**Affiliations:** 1 Key Laboratory of Southwest China Wildlife Resources Conservation (Ministry of Education), China West Normal University, Nanchong 637009, China; 2 Chengdu Institute of Biology, Chinese Academy of Sciences, Chengdu 610213, China; 3 University of Chinese Academy of Sciences, Beijing 101408, China

**Keywords:** Parkinson’s disease, local field potentials, 6-OHDA, motor deficits

## Abstract

Parkinson’s disease (PD) is a neurodegenerative disorder characterized by motor symptoms like tremors and bradykinesia. PD’s pathology involves the aggregation of α-synuclein and loss of dopaminergic neurons, leading to altered neural oscillations in the cortico-basal ganglia-thalamic network. Despite extensive research, the relationship between the motor symptoms of PD and transient changes in brain oscillations before and after motor tasks in different brain regions remain unclear. This study aimed to investigate neural oscillations in both healthy and PD model mice using local field potential (LFP) recordings from multiple brain regions during rest and locomotion. The histological evaluation confirmed the significant dopaminergic neuron loss in the injection side in 6-OHDA lesioned mice. Behavioral tests showed motor deficits in these mice, including impaired coordination and increased forelimb asymmetry. The LFP analysis revealed increased delta, theta, alpha, beta, and gamma band activity in 6-OHDA lesioned mice during movement, with significant increases in multiple brain regions, including the primary motor cortex (M1), caudate–putamen (CPu), subthalamic nucleus (STN), substantia nigra pars compacta (SNc), and pedunculopontine nucleus (PPN). Taken together, these results show that the motor symptoms of PD are accompanied by significant transient increases in brain oscillations, especially in the gamma band. This study provides potential biomarkers for early diagnosis and therapeutic evaluation by elucidating the relationship between specific neural oscillations and motor deficits in PD.

## 1. Introduction

Parkinson’s disease (PD) is a common neurodegenerative condition identified by clinical symptoms including resting tremors, bradykinesia, postural instability, and gait disturbances, along with various non-motor symptoms [1,2]. Pathologically, PD is characterized by the aggregation of abnormal α-synuclein (α-syn) in Lewy bodies, coupled with the loss of dopaminergic neurons in the substantia nigra pars compacta (SNc) and denervation in the corpus striatum (CPu) [3]. Dopamine depletion in PD triggers pathological brain oscillations throughout the cortico-basal ganglia-thalamic network, ultimately leading to the behavioral manifestations of PD syndrome [4,5]. As a type of brain oscillation, local field potentials (LFPs) are recordings of discharges from a cluster of neurons surrounding electrodes, which are mainly formed by extracellular fields generated by synaptic transmembrane currents [6]. These LFPs oscillate at different frequencies and are associated with motor control processes, including sensorimotor integration and voluntary motor preparation [7,8,9]. In rodent PD models, the administration of neurotoxins leads to changes in oscillatory activity in various brain regions, such as the primary motor cortex (M1), subthalamic nucleus (STN), substantia nigra pars (SN), internal globus pallidus (GPi), and pedunculopontine nucleus (PPN) [10,11,12]. Consequently, dynamic changes in LFP activity in these regions are potential biomarkers for PD.

One major area of research on neural oscillations in movement disorders focuses on beta oscillations. Clinical studies on PD patients have identified a significant correlation between the changes in the beta band in the CPu and STN and the severity of the disease [13,14]. From an electrophysiological perspective, increased beta oscillations are believed to result from the increased synchronization of synaptic activity, with highly synchronized neuronal activity carrying less information [15,16]. Consequently, the excessive synchronization of activity in the beta band may limit neural activity to rigid patterns, thus hindering the dynamic changes required for normal motor function [17]. Dopamine replacement therapy and deep brain stimulation (DBS) therapy have been shown to significantly reduce the elevated activity of the beta band typically observed in PD [18]. Comparable changes in beta oscillations have been also found in animal models of PD. For instance, compared with the control rats, rats with 6-OHDA lesions exhibited a significant increase in activity in the beta band (29–36 Hz) in the STN [19,20]. Moreover, previous studies have shown that increased activity in the gamma band is linked to an enhanced movement velocity, yet it exhibits a negative correlation with motor signs in PD [6,17]. Specifically, activity in the low gamma band (31–45 Hz) is positively correlated with the severity of resting tremors [21,22]. In addition, previous studies have indicated a potential link between the activity in the alpha band and gait performance. For example, increased alpha power in the PPN is associated with increased gait speeds, while reduced alpha power is linked to instances of gait freezing [20,23]. Furthermore, the administration of levodopa has been shown to enhance the alpha oscillations in the PPN, leading to improvements in gait function [24]. Conversely, there is a limited number of studies focusing on the low-frequency oscillations detected in individuals with PD, primarily due to the susceptibility of the delta band to motion artifacts, which are frequently filtered out during analysis. In depth electrode recordings, around 50% of the neurons in the substantia nigra pars reticulata (SNr) demonstrated delta oscillations in dopamine-depleted rats [25]. These oscillations may serve as strong indicators of dopamine loss and motor disturbances. However, it remains to determine the relationship between the motor symptoms of PD and transient changes in all LFP bands before and after motor tasks in different brain regions.

The present study aimed to combine behavioral, pathophysiologic, and electrophysiological methods to provide additional insights into the pathogenesis of PD. To this end, we used 6-OHDA lesions to establish a model of PD and validated the model using histological methods. For both the sham mice and 6-OHDA lesioned ones, their behavioral parameters were measured through behavioral assessments. To record brain oscillations, we implanted electrodes in the motor-related brain regions and calculated the power spectra of different frequency bands, as well as their transient differences before and after motor tasks. Based on these results, the relationships between motor symptoms and power spectrum changes were explored.

## 2. Results

### 2.1. Histological Evaluation

**Figure 1:**
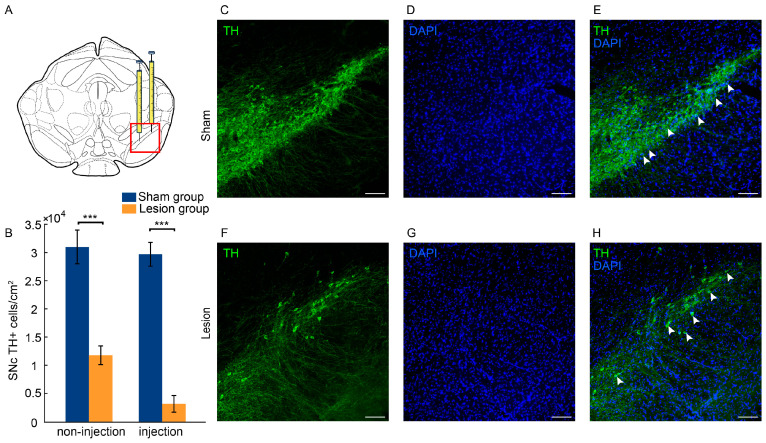
Dopaminergic cell loss in the SNc after 6-OHDA injection. (**A**) Schematic representation of injection sites in the SNc, and the subplots C-H show enlarged pictures of the boxed area. (**B**) The number of TH+ neurons in the SNc for the sham group (n = 10) and the lesion group (n = 10). (**C**–**H**) Immunofluorescence staining of TH+ neurons (green, **C**,**F**), DAPI (blue, **D**,**G**), and a merged image (**E**,**H**) of the injection side of the SNc. The white arrows indicate TH+ neurons in the pictures. The scale bar represents 100 μm. All data are expressed as the mean ± SD. *** *p* < 0.001.

Tyrosine hydroxylase (TH) is a rate-limiting enzyme in the synthesis of catecholamines, including dopamine. Therefore, evaluating the number of TH+ neurons through immunofluorescence detection allows for the quantitative analysis of dopaminergic neuronal degeneration and the assessment of brain tissue damage in 6-OHDA lesioned mice. Compared to the sham group, the unilateral injection of 6-OHDA into the SNc significantly decreased the number of TH+ neurons in the SNc (injection side, Z = −3.808, *p* < 0.001; non-injection side, Z = −3.553, *p* < 0.001; Figure 1A,B). Specifically, the loss of TH+ neurons in the SNc was 89.52% on the injection side and 61.21% on the non-injection side when compared to the sham group. Representative sections were stained to show TH+ neurons in the SNc from both the sham group and lesion group (Figure 1C–H).

### 2.2. Results of Behavioral Tests

**Figure 2:**
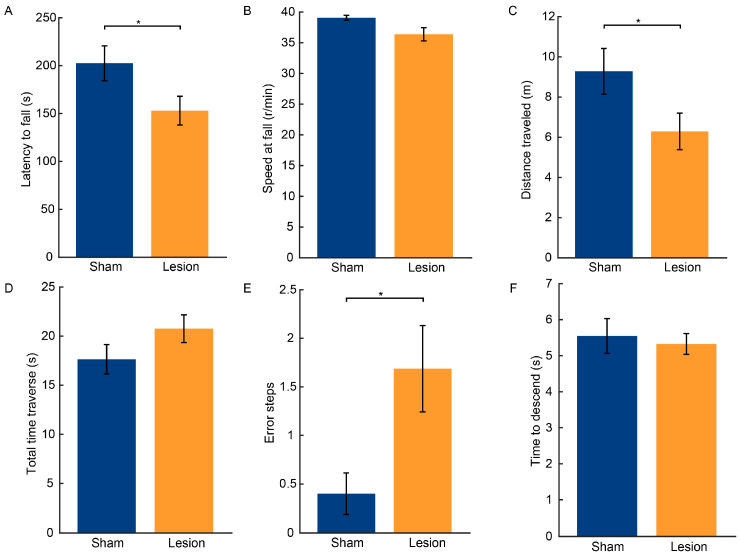
Behavioral performance of mice after 6-OHDA injection. (**A**–**C**) Latency to fall, speed at fall, and total distance traveled in the rotarod test. (**D**–**E**) Total time for traversal and number of hindlimb lapses for the balance beam traversal test. (**F**) Time to descend from top to base in the pole test. n = 16 for the lesion group and n = 15 for the sham group. All data are expressed as the mean ± SD. * *p* < 0.05.

The results of the rotarod test indicated that the motor coordination of the lesion group was adversely affected by the injection of 6-OHDA. In comparison to the sham group, the lesion group exhibited a significant decrease in the latency to fall from the rotarod (t = −2.115, *p* < 0.05, Figure 2A) and a significant reduction in the total distance traveled (t = −2.072, *p* < 0.05, Figure 2C). Moreover, the lesioned group typically could not stay on the rod for the entire 300 s test period, falling off before the speed reached 40 rpm. However, there was no significant difference in the speed at the time of fall between the two groups (Z = −2.494, *p* > 0.05, Figure 2B).

The balance and coordination of the mice’s hindlimbs were evaluated through the balance beam traversal test, as rodents typically rely on their forelimbs for intricate movements. The results showed that the lesion group required a longer time to traverse the balance beam, but the difference between the two groups did not reach statistical significance (t = 1.523, *p* > 0.05, Figure 2D). However, the lesion group exhibited a significantly increased incidence of hindlimb error steps (Z = −2.494, *p* < 0.05, Figure 2E).

The pole test assesses the grasping capabilities of the forelimbs and the overall motor coordination of mice. The test revealed no significant differences in the descent latency between the lesion group and the sham group (Z = −0.395, *p* > 0.05; Figure 2F). This suggests that the unilateral 6-OHDA injection did not significantly impair the grasping capabilities or fine motor skills of the mice.

**Figure 3:**
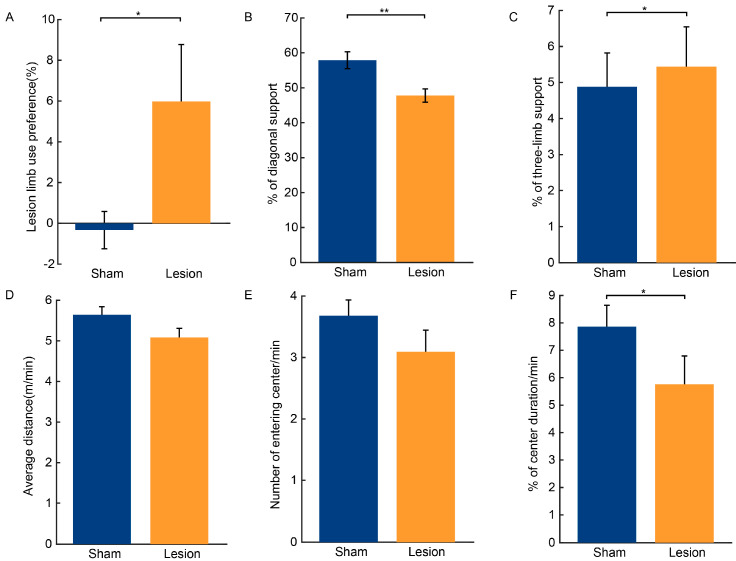
Limb symmetry, gait impairment, and spontaneous locomotion after 6-OHDA injection. (**A**) Percentage of forelimb use on the injection side in the rearing cylinder test. n = 16 for the lesion group and n = 12 for the sham group. (**B**,**C**) Percentage of diagonal support and three-limb support during locomotion. (**D**–**F**) Average distance moved, number of entries into the central area, and percentage of time spent in the central area in the open field test. For pictures (**B**–**F**), n = 16 for the lesion group and n = 15 for the sham group. All data are expressed as the mean ± SD. * *p* < 0.05, ** *p* < 0.01.

The rearing cylinder test is often used to measure spontaneous locomotion and limb use asymmetry in mice. The results showed that the lesion group exhibited significantly increased forelimb asymmetry and a preference for the use of the injection-side forelimb (t = 2.141, *p* < 0.05, Figure 3A).

The 6-OHDA lesion resulted in significant deficits in gait stability. There was a significant decrease in the use of diagonal support throughout the gait cycle, which was compensated for by the significantly increased reliance on three-limb support (diagonal support, t = −3.277, *p* < 0.01; three-limb support, t = 2.763, *p* < 0.05, Figure 3B,C).

The open field test revealed that the spontaneous locomotor capacity of the lesion group experienced a negligible impact, as evidenced by the average distance traversed in the open field (t = −1.332, *p* > 0.05, Figure 3D) and the percentage of the mice’s entries into the central zone (t = −1.884, *p* > 0.05, Figure 3E). However, the duration spent in the central area by the lesion group was significantly reduced compared to the sham group (Z = 2.095, *p* < 0.05, Figure 3F).

### 2.3. Dynamic Changes in LFP Power Spectra Following Dopaminergic Lesion across Resting and Walking Phases

**Figure 4:**
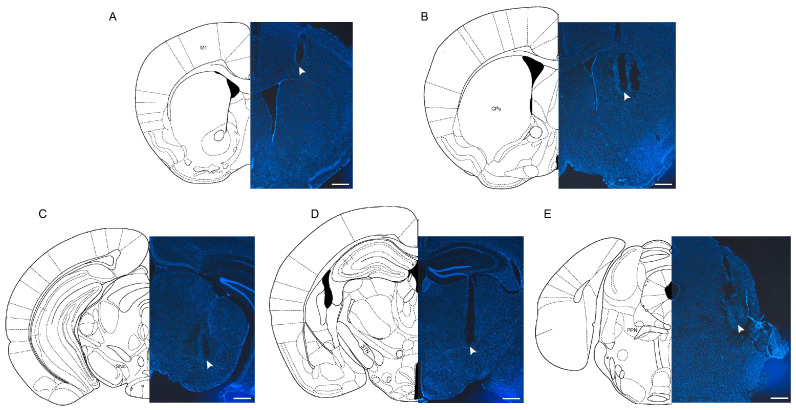
Verification of electrode placement. (**A**) Primary motor cortex (M1). (**B**) Corpus striatum (CPu). (**C**) Substantia nigra pars compacta (SNc). (**D**) Subthalamic nucleus (STN). (**E**) Pedunculopontine tegmental nucleus (PPN). Arrows indicate electrode sites. The scale bar represents 500 μm.

Electrodes designated for LFP recording were implanted in the M1, CPu, STh, SNc, and PPN. Upon comparison with the Mouse Brain Atlas [26], it was verified that the electrodes were implanted in the correct locations as they closely matched those depicted in the atlas. The results of the DAPI staining of these five brain regions are shown in Figure 4.

**Figure 5:**
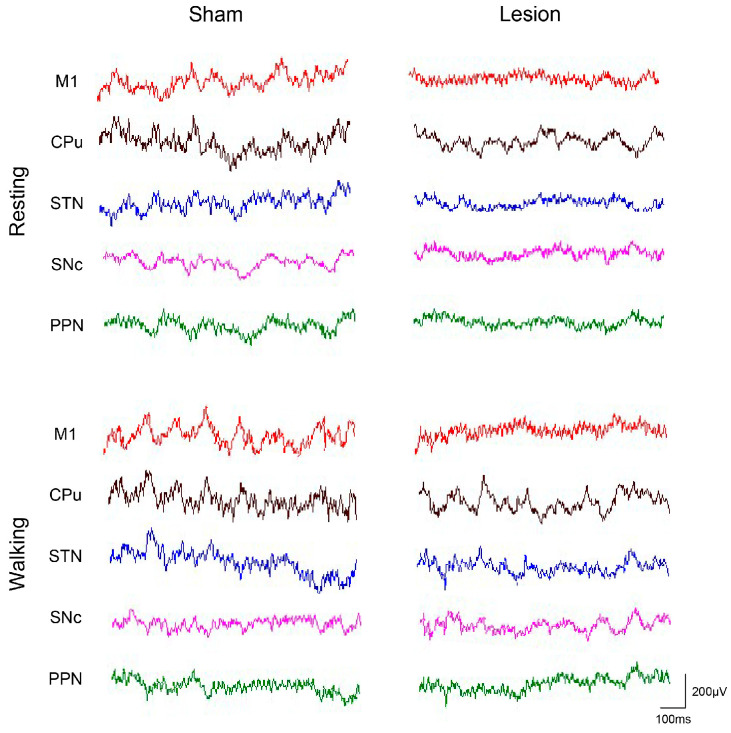
Typical LFP waveforms recorded from the M1, CPu, STN, SNc, and PPN during resting and walking states for both sham and lesion mice.

**Figure 6:**
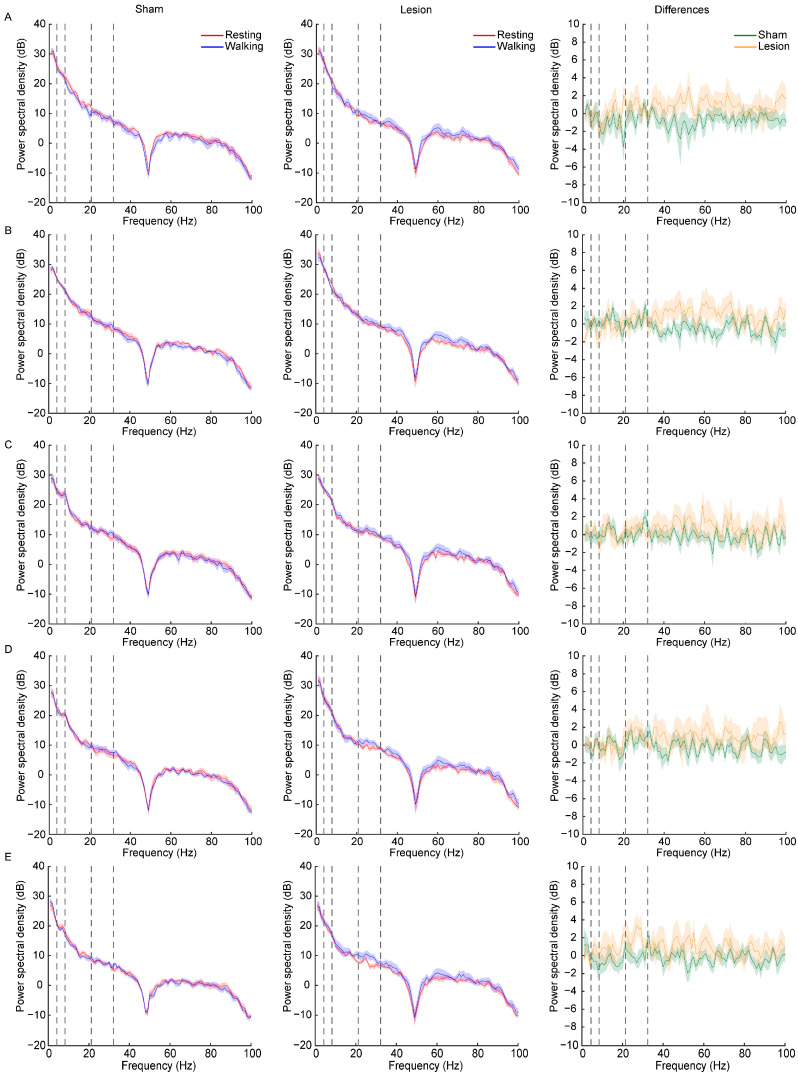
Power spectral density of LFPs recorded for the M1 (**A**), CPu (**B**), STN (**C**), PPN (**D**), and SNc (**E**). Left column shows average power spectral density of sham group during resting (red) and walking (blue) states for each brain region; middle column indicates average power spectral density of lesion group during resting (red) and walking (blue) states for each brain region; right column illustrates differences in power spectral density between walking and resting states in sham group (green) and lesion group (orange). All data are expressed as mean ± SEM. The dotted lines divide the frequency range into the delta (1–4 Hz), theta (4–8 Hz), alpha (8–21 Hz), beta (21–32 Hz), and gamma (32–100 Hz) bands.

**Figure 7:**
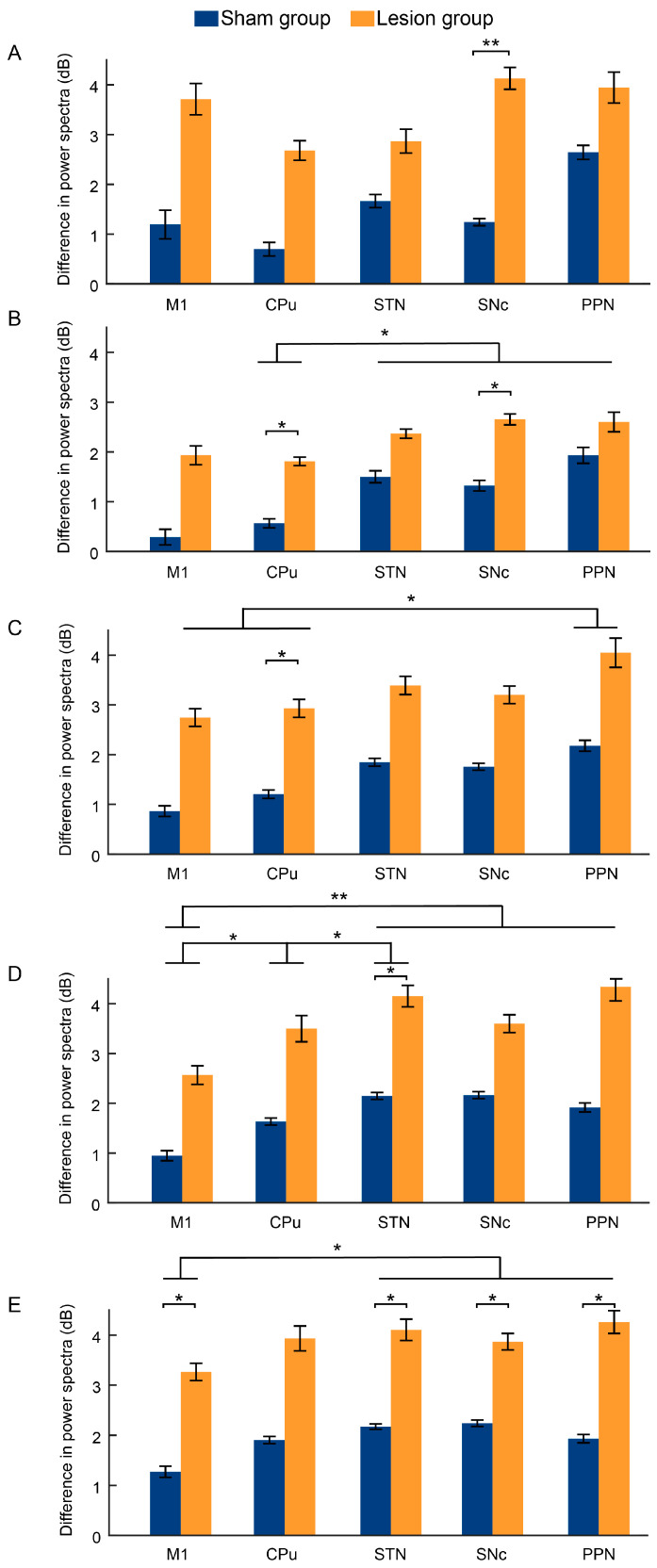
Difference in power spectra of various bands of LFP, including delta (1–4 Hz) (**A**), theta (4–8 Hz) (**B**), alpha (8–21 Hz) (**C**), beta (21–32 Hz) (**D**), and gamma (32–100 Hz) (**E**) bands. n = 10 for each group. All data are expressed as the mean ± SD. * *p* < 0.05, ** *p* < 0.01.

LFP data were acquired from the mice during the periods of resting and walking in the rotarod test, with representative LFP waveforms (Figure 5) and the changes in the average power spectral density across different frequency bands for the two groups (Figure 6) shown below. In the power spectra of the different groups, the statistical results showed that the differences in the power spectra between the walking and resting periods exhibited a significant increase in the lesion group compared with the sham group for the delta and gamma bands (Figure 7A,E, Table 1) and a marginally significant increase for the theta, alpha, and beta bands (Figure 7B–D, Table 1). Additionally, compared to the sham group, the lesion group exhibited a significant increase in activity within the delta band in the SNc (t = 3.112, *p* < 0.01), within the theta band in the CPu (t = 2.506, *p* < 0.05) and SNc (t = 2.158, *p* < 0.05), within the alpha band in the CPu (t = 2.394, *p* < 0.05), within the beta band in the STN (t = 2.209, *p* < 0.05), and within the gamma band in the M1 (t = 2.424, *p* < 0.05), STN (t = 2.210, *p* < 0.05), SNc (t = 2.312, *p* < 0.05), and PPN (t = 2.396, *p* < 0.05), as shown in Figure 6 and Figure 7.

In the power spectra of the different brain regions, there was no significant difference between the different channels in the delta band (*p* > 0.05, Figure 7A and Table 1). For the theta band, the STN, SNc, and PPN exhibited significantly higher power spectra than those in the CPu (all *p* < 0.05, Figure 7B and Table 1). In the alpha band, the power spectra in the PPN were marginally significantly higher than those in both the M1 and CPu (all *p* < 0.05, Figure 7C and Table 1). Furthermore, the power spectra in the beta band for the CPu, STN, SNc, and PPN were significantly higher than those for the M1, and those for the STN were significantly higher than those for the CPu (all *p* < 0.05, Figure 7D and Table 1). In addition, the power spectra in the beta band for the STN, SNc, and PPN were significantly higher than those for the M1 (all *p* < 0.05, Figure 7E and Table 1).

## 3. Discussion

### 3.1. Loss of Dopaminergic Neurons in the SNc and Motor Function Impairments Resulting from 6-OHDA

On the one hand, the present results demonstrate that unilateral 6-OHDA injection into the SNc cause the severe loss of dopaminergic neurons in mice. Specifically, compared with the sham group, the loss of TH+ neurons in the SNc was 89.52% on the injection side and 61.21% on the non-injection side in the lesion group. These findings are consistent with previous studies showing that unilateral 6-OHDA injection into the SNc results in significant damage to the nigrostriatal pathway on the injection side [27,28]. Moreover, the unilateral SNc injection of 6-OHDA alters the monoamine (e.g., DA and 5-HT) metabolite levels in the brain, potentially causing bilateral damage [29]. Our findings are consistent with this previous study, indicating that the observed decrease in DA neurons on the non-injection side may be part of a broader, bilateral response to the lesion.

On the other hand, unilateral 6-OHDA injection into the SNc leads to behavioral deficits, including in locomotor activity and coordination, serving as a sensitive indicator of degenerative changes in dopaminergic neurons. For example, compared with the sham mice, the 6-OHDA lesioned mice exhibited a significantly shorter latency to fall and distance traveled in the rotarod test. Moreover, the 6-OHDA lesioned mice exhibited hindlimb motor impairments and a preference for the use of the forelimb ipsilateral to the injection side in the cylinder test, reflecting gait asymmetry akin to that observed in PD patients [30]. Additionally, the 6-OHDA lesioned mice demonstrated decreased diagonal support but increased three-limb support in the gait tests. In PD patients, the shuffling gait pattern is characterized by an increased duration of the gait cycle with both feet in contact with the ground, resulting in an increased double support phase to compensate for the fear of falling or postural instability [31,32,33]. Quadrupedal animals share similar gait characteristics with humans. In rodents, the pairs of forelimbs or hindlimbs on opposite sides of the body exhibit left–right alternation, with diagonal forelimbs and hindlimbs moving simultaneously during walking [34,35]. In this gait pattern, at least two feet are in contact with the ground at any given time, ensuring stability. Typically, there is also a period when three limbs support the body simultaneously. In PD mice, the decrease in diagonal support indicates increased difficulty in maintaining balance and stability while walking [36,37]. This implies that the mice struggle to coordinate their limb movements during the gait cycle. Meanwhile, the increase in three-limb support found in the 6-OHDA lesioned mice indicates a need for more support points to maintain balance. This phenomenon reflects a decline in motor ability in the mice, which were compensating for the loss of balance and coordination by increasing the number of limbs in contact with the ground. These results collectively demonstrate a loss of motor function in the 6-OHDA lesioned mice, particularly in terms of limb coordination and movement abilities.

### 3.2. Transient Changes in LFP Bands after 6-OHDA Lesion

Human movement and gait are complex, requiring a continuous and coordinated information flow between specialized brain regions, known as the motor network. This network includes several cortical areas (such as the premotor area, supplementary motor area, and parietal cortex), the basal ganglia, the midbrain locomotor region (MLR), the thalamus, the cerebellum, and the central pattern generators (CPGs), i.e., spinal neuron circuits that control the basic rhythms and patterns of motor neuron activation during movement [38]. Each of these structures plays a specific role in motor and gait control. Here, we recorded the LFP from the M1, CPu, STN, SNc, and PPN in the sham and 6-OHDA lesioned mice during resting and walking periods. The results showed that the transient changes in all LFP bands before and after the motor tasks in the entire cortex–basal ganglia circuit and the midbrain PPN region exhibited varying degrees of change.

In studies involving human patients, the analysis of the delta frequency band is often overlooked due to detection difficulties and the susceptibility to low-frequency noise and motion artifacts. We found a significant increase in delta power in the SNc during movement in the 6-OHDA lesioned mice, which is consistent with previous studies showing a marked increase in striatal delta power in DAT-KO mice treated with the TH inhibitor α-methyl-p-tyrosine (AMPT) [39]. However, other previous studies have reported a decrease in the low-frequency LFP power, including the delta band, in the dorsal striatum (dSTR) and STN during both rest and movement [40,41]. This discrepancy may be attributed to various factors, such as the modeling methods, the extent of dopamine damage, and the motor or cognitive state of the animal models, necessitating further investigation.

The present results showed a significant increase in the theta band (4–8 Hz) in the CPu and SNc. However, a similar increase in theta power was not observed in PD rats [42]. In contrast, previous studies have reported an increase in the theta rhythm in the SNr during L-DOPA-induced dyskinesia (LID) in rats, along with a non-significant tendency for enhanced theta oscillatory activity in the dSTR [42]. Dopaminergic drug-induced dyskinesia in PD patients has also been found to be associated with an increase in the 4–10 Hz frequency band [43]. It has also been shown that low-frequency oscillatory activity (3–6 Hz) in the basal ganglia, including the STN, medial globus pallidus, and lateral globus pallidus, is strongly associated with limb tremors in MPTP monkeys and patients with PD in the “off” state [44,45]. Taken together, these findings indicate that an increase in lower-frequency oscillations (below 10 Hz) can be observed in hyperkinetic states, such as abnormal involuntary movement and resting tremors.

Our study also found a significant increase in the alpha band power (8–21 Hz) in the CPu. Previous research has linked alpha band activity with limb movement. Specifically, compared to the resting state, PD patients exhibited a marked increase in the bilateral alpha band power in the STN and GPi during rapid punching tasks [46]. During movement, including free walking or an imagined gait, there is an increase in the alpha band power in the PPN, which correlates with the gait speed [47,48]. In patients with dystonia, walking on a treadmill resulted in a significant increase in power across the theta, alpha, and gamma bands in the GPi [49]. Conversely, during gait freezing, there was a decrease in the alpha band power [24,47]. These findings demonstrate that neural oscillations in the alpha band are associated with ongoing limb movement.

In the frequency range of 21–32 Hz, the 6-OHDA lesioned mice exhibited enhanced beta oscillations in the STN during walking. Currently, although the functional and pathological role of beta oscillations remains controversial, such oscillations have been detected in humans and PD animal models in most brain regions of the cortico-basal ganglia circuit, including the STN, the globus pallidus (GP), SNr, the thalamus, and the motor cortex [50,51,52]. Synchronization in the beta band has been hypothesized to be essentially antikinetic in nature and pathophysiologically relevant to bradykinesia [53]. Many studies have observed an increase in beta power in the STN in PD patients, with a decrease during movement preparation and execution, followed by an increase at the conclusion of movement [13,54]. After treatment with L-DOPA, the beta power decreases [55]. This contrasts with our study findings, but it is noteworthy that differences in frequency band selection across studies may explain the heterogeneity in the results among different studies. A previous study that recorded the LFP in rats revealed a significant increase in the beta frequency range in PD rats during walking in the M1 (15–24 Hz), STN (13–28 Hz), and PPN (14–22 Hz) [56]. Another study indicated differential effects on beta band activity in the affected hemisphere in PD rats, with the low beta frequency range (12–25 Hz) and high beta frequency range (25–40 Hz) showing varying degrees of influence [57]. During walking, the affected hemisphere’s SNr exhibited prominent high-frequency beta activity (27–34 Hz); in contrast, the low beta power (12–25 Hz) was reduced in both lesioned and non-lesioned hemispheres in PD rats [57]. Furthermore, research has shown that the power in the high beta frequency range (25–40 Hz) increases with varying movement speeds in rodents [7]. In summary, it can be seen that as the movement activity increases or decreases, the power of low-frequency and high-frequency oscillations changes in opposite directions. This may reflect the different functional states of activity within the motor-related neural control systems.

The present results show that the 6-OHDA lesioned mice, during walking, exhibited a significant increase in gamma power in all brain regions, with the exception of the CPu. Gamma band activity is generally considered prokinetic, in contrast to beta band activity, as it is associated with the initiation of movement [58]. Increased activity in the gamma band (60–80 Hz) was observed during treatment with L-DOPA and was associated with the development of L-DOPA-induced motor disorders [58]. Under pathological conditions, increased beta activity in the STN during movement may contribute to the bradykinetic symptoms experienced by many patients. Increasing gamma band activity may serve as a compensatory mechanism within the neural network to facilitate movement under beta band antikinetic conditions [59].

Furthermore, we found that the power spectra of brain regions such as the STN, SNc, and PPN were generally higher than those of the M1 and CPu across all frequency bands. This result is consistent with the shared involvement of these brain regions in complex motor processes, including gait–postural adjustments and the initiation and maintenance of movement. Previous studies have found that the basal ganglia, including the CPu and STN, processes different movement parameters, such as complexity and frequency. Specifically, enhanced fMRI signals were observed primarily in the STN during complex and high-frequency movement tasks, while the CPu showed increased signals regularly during tasks with a lower movement frequency and less complexity [60]. The rotarod test requires mice to passively and quickly adjust their steps to prevent falling and maintain body balance, rather than moving freely. Therefore, this more complex motor task may explain the differences in the power spectra between the STN and CPu. In addition, during walking, the alpha activity in the PPN was closely associated with gait performance, which may be related to the higher power spectra of the PPN in the alpha band [61]. However, due to the complex interactions among brain regions, future research should further explore the relationship between brain region functions, heterogeneous neuronal populations, and electrophysiological properties to gain a deeper understanding of the role of oscillatory activities in different brain regions and frequency bands in motor control.

While our study revealed significant transient LFP alterations across various bands during movement in lesioned mice, two limitations must be acknowledged. First, we did not record neural activity from the non-injection side. Given that unilateral injections of 6-OHDA in SNc can lead to bilateral damage, it is crucial for future studies to investigate the relationship between transient LFP alterations and the extent of neural damage. Second, the present work did not assess whether the properties of the LFP in the PD model mice could return to the baseline levels following treatment with anti-PD drugs, such as L-DOPA. Further research is warranted to elucidate the effects of these pharmacological interventions on the LFP properties.

## 4. Materials and Methods

### 4.1. Animals

The experiments were conducted using adult male C57BL/6 mice procured from Chengdu Dossy Experimental Animals Co., Ltd., Chengdu, China. The animals were housed in an individually ventilated caging system (IVC; ZJ-4, Fengshi, Suzhou, China) with a 12 h/12 h light cycle (lights on at 8:00 a.m.), and the ambient temperature and relative humidity were maintained at 22 ± 1 °C and 65% ± 5%, respectively. Water and food were available ad libitum. Twenty mice were randomly divided into two groups: the sham group and lesion group. All behavioral and electrophysiological experiments were performed on mice aged 8–14 weeks.

### 4.2. Surgery

Both unilateral 6-OHDA lesions and electrode implantations were performed on the mice placed in a stereotaxic apparatus (51673U, Stoelting, Wood Dale, IL, USA) under isoflurane (1–3%) anesthesia. All coordinates were presented with anterior–posterior (AP) and medial–lateral (ML) distances measured from the bregma and dorsal–ventral (DV) distances referenced to the dural surface.

#### 4.2.1. Unilateral 6-OHDA Lesions

To protect the noradrenergic and serotoninergic systems, the mice received desipramine (0.625–0.75 mg, i.p.) 30 min prior to unilateral 6-OHDA injection. The 6-OHDA (12 mg/mL, Sigma, Darmstadt, Germany) was dissolved in sterile saline (0.9%) containing ascorbic acid (0.2%). Given the relatively narrow morphology of the SNc, two specific sites in this nucleus were used for unilateral lesions: AP −3.16 mm, ML ±1.25 mm, DV −4.3 mm; and AP −3.16 mm, ML ±1.5 mm, DV −4.1 mm. Injections were administered at 100 nl per site (40 nl/min) using microliter syringes (NRS75 RN 5.0 μL (33/20/3), Hamilton, Reno, Nevada, Switzerland). Then, the syringe needle remained in situ for 10 min, after which it was gradually withdrawn. The sham mice received infusions of the vehicle only. Following surgery, the animals were given a minimum recovery period of two weeks before behavioral testing.

#### 4.2.2. Electrode Implantation

Five formvar-insulated nichrome wires (0.8 mm in diameter) were implanted in the primary motor cortex (M1), corpus striatum (CPu), subthalamic nucleus (STN), SNc, and pedunculopontine tegmental nucleus (PPN) on the injection side. The coordinates were AP +0.98 mm, ML ±1.50 mm, and DV −1.50 mm for M1; AP +0.38 mm, ML ±2 mm, and DV−3.5 mm for CPu; AP −1.82 mm, ML ±1.50 mm, and DV −4.4 mm for STN; AP −3.16 mm, ML ±1.25 mm, and DV −4.4 mm for SNc; and AP −4.72 mm, ML ±1.28 mm, and DV −3.45 mm for PPN. The reference electrode was implanted above the cerebellum (AP −6.5 mm and ML 0 mm). The wires were fixed to the skull with dental cement. After surgery, the mice were returned to the home cage. They were allowed to recover for 7 days before subsequent experiments were performed.

### 4.3. Behavioral Tests

#### 4.3.1. Rotarod Test

The rotarod test was performed to evaluate locomotor activity and coordination, serving as a sensitive indicator of degenerative changes in dopaminergic neurons. In the rotarod test, the mice are required to move on a rotating cylindrical rod. Before the experiment, the mice were transferred to the testing room for a period of 30 min to allow for acclimation. Subsequently, a two-day training regimen was implemented: on the first day, the mice underwent training on the rotarod apparatus (XR-6C, Xinruan, Shanghai, China) at a low, constant speed of 5 rpm until they could maintain stability on the rotarod for 2 min without falling; on the second day, training commenced at a constant speed of 10 rpm, followed by adaptation to a constant acceleration mode (from 4 to 40 rpm). During the test on the third day, the constant acceleration mode was employed. The mice had to coordinate their movements and maintain their balance to ensure that they remained on the rod without falling off. If the mice fell, the latency, speed, and total distance traveled in the current trial were automatically recorded. Each mouse underwent three trials, with a minimum interval of 15 min between trials.

#### 4.3.2. Pole Test

The mice were positioned head down atop an upright wooden pole (50 cm in height, 1 cm in diameter) affixed to a square base, and the duration required for the mice to descend from the top to the bottom of the pole was recorded. Each mouse underwent a two-day training regimen comprising five trials per day. On the third day, the mice were subjected to three test trials, with the pole meticulously cleaned with alcohol after each trial.

#### 4.3.3. Beam Traversal Test

This test requires the mice to traverse a 1-m-long balance beam (consisting of four segments, each 0.25 m long and 3.5 cm, 2.5 cm, 1.5 cm, and 0.5 cm wide) without any assistance. During the two-day training period, the mice were positioned at the wider end of the beam and manually guided to the opposite end, which was connected to a darker cardboard box. For a given subject, successful training was achieved when the animal traversed the beam three times successfully. On the third day, the mice needed to traverse the balance beam without any assistance. The time that elapsed from the mouse’s placement to crossing the beam and touching the endpoint with one forelimb was recorded, along with the number of hindlimb missteps during traversal.

#### 4.3.4. Open Field Test

The open field test is used to detect spontaneous locomotor activity in mice. The mice were transferred to a behavioral chamber for a 2 h acclimatization period. Upon placement in the center of the open field (measuring 40 cm × 40 cm), the activities of the mice were recorded under dim light during the dark phase for a duration of 10 min. Subsequent analysis was conducted using the Cineplex software v3.6.0 (Plexon, Dallas, TX, USA) to quantify parameters including the total distance traveled, the number of entries into the central area, and the duration of residence in the central area. The arena was cleaned with 75% alcohol at the end of each trial.

#### 4.3.5. Rearing Cylinder Test

The mice were placed in a glass cylinder (15 cm in diameter and 20 cm in height) with a 40-lux white light provided directly above. Two mirrors were placed next to the cylinder to ensure that the camera could clearly capture the fine movements of the mice’s forelimbs in every direction for 5 min. The number of touches made by the lesion-side forelimb alone, the intact-side forelimb alone, and both forelimbs simultaneously touching the wall was recorded. Subsequently, an asymmetry ratio was calculated as an index of voluntary forelimb use according to the formula (number of touched by the injection side − number of touched by the non-injection side)/(number of touched by the injection side + number of touched by the non-injection side + number of touched by both sides) × 100%. The cylinder was cleaned with 75% alcohol at the end of each trial.

#### 4.3.6. Gait Analysis

Gait analysis was performed using a visual gait analysis system (VisuGait, Xinruan, Shanghai, China) to collect and analyze the gait parameters of the mice. The mice underwent a two-day training period to familiarize themselves with the track. Each mouse underwent three trials, and the runway was cleaned with 75% alcohol after each trial.

### 4.4. Electrophysiological Recordings and Data Analysis

At least three weeks after lesion creation, the LFPs were recorded using a multi-channel data acquisition system (RM6280, Chengyi, Sichuan, China) under two conditions: a resting phase (5 min), during which the rotarod remained motionless, and a walking phase (5 min), with the rotarod kept rotating at a constant speed of 5 rpm. The sampling frequency was set to 1000 Hz.

The LFP signals were filtered offline using a band-pass filter of 0.5–80 Hz. We extracted artifact-free 10 s EEG segments corresponding to the resting and walking phases for both the 6-OHDA lesioned mice and sham mice and then divided these segments into 1 s epochs. For each brain region, the power spectrum was acquired for each band, i.e., delta (1–4 Hz), theta (4–8 Hz), alpha (8–21 Hz), beta (21–32 Hz), and gamma (32–100 Hz) [62]. The differences between the power spectra for the walking phase and those for the resting phase were investigated via statistical analysis.

### 4.5. Histology and Immunohistochemistry

Following the completion of the LFP recording, each brain region containing the implanted electrodes underwent current-induced damage for 8 s using a programmed stimulator (YC-2, Chengyi, Chengdu, Sichuan) at a current intensity of 0.4 mA to identify the position of the electrode tip. Furthermore, dopaminergic neuronal damage in PD mice was assessed via the quantitative analysis of TH+ neurons. To do this, the mice were cardiac-perfused with 1 mM phosphate buffer solution (PBS) and pre-fixed with 20–50 mL of 4% paraformaldehyde (PFA). The brains were extracted, post-fixed for 24 h, and immersed in sucrose (15–30% *w*/*v* in 4% PFA) before being sectioned. The brains were cut into 30 μm coronal sections with a cryostat microtome (CM1850, LEICA, Nussloch, Germany) and collected in six-well cell culture plates filled with PBS.

All following washing steps were performed in 0.1% (*w*/*v*) Triton PBS and the sections were rinsed three times for 5 min each. After extensive washing, the sections were permeabilized with 0.5%(*w*/*v*) Triton PBS for 15 min. The sections were then subjected to three washes and blocked and permeabilized for 1 h at room temperature in a PBS solution containing 3% bovine serum albumin (BSA). The sections were incubated in the primary antibody (Rabbit Anti-Tyrosine Hydroxylase Antibody; Millipore, Burlington, MA, USA, 1:1000) for a minimum of 36–48 h at 4 °C. Subsequently, the sections were washed and incubated for 2 h in a blocking solution containing a secondary antibody to reveal TH (Donkey Anti-Rabbit IgG H&L; Alexa Fluor^®^ 488; pre-adsorbed, Abcam, Cambridge, UK, 1:1000). Nuclei were stained with DAPI, which appeared in the blue channel after washing. The brain sections were then covered with a glass coverslip and stored at 4 °C before observation.

### 4.6. Cell Counting

Images were acquired using a fluorescence microscope (NE900, Nexcope, Ningbo, China) at 20× magnification. The brain regions were identified based on the Mouse Brain Atlas [26]. The quantification of TH+ cells was performed on ten mice per group (sham group and lesion group). Specifically, three slices were randomly selected from the rostral to caudal regions of the SNc for each mouse. The brain regions were identified according to the Mouse Brain Atlas [26], and the areas designated for quantification were delineated by outlining the boundaries of the SNc. Cell counting of all TH+ cells was conducted using the ImageJ software, focusing on the neuronal morphology and DAPI-stained nuclei within this region. The neuron density was calculated for each slice, with the average density across the three brain slices employed for further statistical analysis.

### 4.7. Statistical Analysis

The normality of distributions and the homogeneity of variance for all values were evaluated using the Shapiro–Wilk W test and Levene’s test, respectively. For the number of TH+ cells and behavioral data meeting the statistical assumptions, the independent samples *t*-test was used to examine intergroup differences. For behavioral data that did not meet the statistical assumptions even after transformation (log or square root), the Mann–Whitney U test was used to compare the intergroup differences. For the power spectra of each LFP band, two-way repeated-measures ANOVAs with the factors “group” (sham group and lesion group) and “brain region” (the five brain regions) were utilized. Greenhouse–Geisser correction was employed when the assumption of sphericity was violated. If the ANOVAs returned a significant difference, the data were further tested for multiple comparisons using the least significant difference test. Statistical analysis was performed using the SPSS software (v23.0, IBM, Chicago, IL, USA), with a significance level set at *p* < 0.05, and *p* ≥ 0.05 and <0.1 were deemed marginally significant [63].

## 5. Conclusions

This study provides insights into the relationship between transient changes in brain oscillations in the basal ganglia network and motor dysfunction after dopamine loss resulting from a 6-OHDA -lesion. The results of the behavioral tests showed that the 6-OHDA lesioned mice had significantly reduced motor coordination and abnormal limb movements. Neurophysiologically, there was a marked increase in power within the delta, theta, alpha, beta, and gamma bands in the basal ganglia–cortical loop and its output region, the midbrain PPN, under lesioned conditions. In other words, these results show that the motor symptoms of PD are accompanied by significant transient increases in brain oscillations, especially in the gamma band. This study provides potential biomarkers for early diagnosis and therapeutic evaluation by elucidating the relationship between specific neural oscillations and motor deficits in PD. These findings raise interesting questions about the functional significance of different frequency bands in the basal ganglia–cortical loop and midbrain regions, providing a foundation for further investigation into these phenomena. Further studies combining electrophysiological methods, neural tracing, and behavioral tasks will be needed to determine how a 6-OHDA lesion in a given brain region results in transient changes in brain oscillations before and after motor tasks in almost all brain regions related to movement.

## Figures and Tables

**Table 1:** Results of ANOVA for power spectra for different bands.

Band	Factor	F	*p*-Value	Partial η^2^	LSD
Delta	group_(1,18)_^1^	4.516	0.048	0.201	Lesion > Sham ^2^
	channel_(4,72)_	1.608	0.204	0.082	NA ^3^
	group*channel_(4,72)_	0.635	0.639	0.034	NA
Theta	group_(1,18)_	4.191	0.056	0.189	Lesion > Sham
	channel_(4,72)_	3.016	0.048	0.144	PPN/SNc/STN > CPu
	group*channel_(4,72)_	0.420	0.794	0.023	NA
Alpha	group_(1,18)_	3.999	0.061	0.182	Lesion > Sham
	channel_(4,72)_	3.457	0.050	0.161	PPN > CPu/M1
	group*channel_(4,72)_	0.095	0.984	0.005	NA
Beta	group_(1,18)_	4.425	0.050	0.197	Lesion > Sham
	channel_(4,72)_	6.491	0.001	0.265	PPN/SNc/STN > M1; CPu > M1; STN > CPu
	group*channel_(4,72)_	0.708	0.589	0.038	NA
Gamma	group_(1,18)_	5.804	0.027	0.244	Lesion > Sham
	channel_(4,72)_	3.616	0.023	0.167	PPN/SNc/STN > M1
	group*channel_(4,72)_	0.432	0.785	0.023	NA

^1^ Numbers in parentheses are degrees of freedom. ^2^ The symbol “>” signifies that the power spectra on the left side are significantly larger than those on the right. No significant difference is observed among the conditions on the same side of “>”. Lesion: Lesion group; Sham: Sham group. ^3^ NA, not applicable. The asterisk (*) in the table indicates the interaction between the two variables.

## Data Availability

Data are available in a publicly accessible repository: https://github.com/myleafmavis/PD-brain-Oscillatory-and-behavior-Database.git (accessed on 31 July 2024).
